# Analysis of Learning Motivation and Burnout of Malaysian and Chinese College Students Majoring in Sports in an Educational Psychology Perspective

**DOI:** 10.3389/fpsyg.2021.691324

**Published:** 2021-12-29

**Authors:** Ruilin Xu, Qinglei Wang, Ngien Siong Chin, Eng Wah Teo

**Affiliations:** ^1^Centre for Sport and Exercise Sciences, University of Malaya, Kuala Lumpur, Malaysia; ^2^School of Physical Education, Zhengzhou University, Zhengzhou, China; ^3^Institute of Teacher Education Batu Lintang Campus, Kuching, Malaysia

**Keywords:** educational psychology, sports major, learning motivation, learning burnout, college students

## Abstract

The purposes are to deepen the understanding of the correlation between learning motivation and learning burnout and thereby stimulate the learning motivation of college students. According to the theory of educational psychology, mechanism of learning motivation, and causes of learning burnout are analyzed. The learning motivation and learning burnout of college students majoring in sports are taken as the breakthrough point. The learning motivation and learning burnout situation of Chinese and Malaysian students majoring in sports are surveyed and compared through questionnaires. In addition, Chinese students majoring in sports are taken as examples to analyze the impact of learning motivation on learning burnout. The correlations between various dimensions are analyzed to determine the impact of learning motivation on learning burnout. The total learning motivation scores of students from the Sport School of Zhengzhou University and the University of Malaya Centre for Sport and Exercise Sciences are 122.3 ± 22.4 and 140.2 ± 23.6, respectively, and their average scores for each question are 3.60 and 4.07, respectively. The total learning burnout scores of students from the Sport School of Zhengzhou University and the University of Malaya Centre for Sport and Exercise Sciences are 58.2 ± 8.95 and 53.6 ± 7.34, respectively. The learning motivation of Malaysian college students majoring in sports is slightly stronger than Chinese students. Compared with Malaysia, the learning burnout of college students majoring in sports in China is extra apparent, mainly exhibited in the two dimensions of depression and a low sense of achievement. The learning motivation and learning burnout of college students majoring in sports are negatively correlated; that is, the stronger the learning motivation, the weaker the learning burnout; on the contrary, the weaker the learning motivation, the more severe the learning burnout. In conclusion, learning burnout of college students can be reduced by correcting and stimulating their learning motivation and improving their learning self-efficacy.

## Introduction

The world is in a globalized development situation. Under the influence of such an international environment, all countries will encounter the problem of effectively improving the teaching effect of college students. The university is a very significant stage of knowledge learning. Students have to acquire in-depth knowledge of various professional sports skills and undergo a psychological experience in the transforming process of the school to society (Vasconcellos et al., 2020). During their university lives, the psychology of students gradually matures, their enthusiasm for learning is continuously improved, and their dialectical thinking ability is increasingly enhanced. The strengthening of the non-intellectual factors of college students is reflected in learning motivation. This inner motivation will drive individuals to work toward their goals and play an influential role in human development (Chang et al., 2016). The unique feature of the university stage is that students begin to recognize and face employment pressure. This factor will affect the physical and mental development of college students, and their learning motivation may gradually shift or disappear (Sparks et al., 2017; Bechter et al., 2018). Learning motivation is not a single psychological activity but a dynamic system composed of multiple psychological factors. When students are learning uninterested knowledge under a compulsory condition, the lack of positive motivation will make students feel bored with the behavior of learning; that is, the phenomenon of learning burnout appears.

The sports major is a specialized profession for cultivating all kinds of sports talents. It teaches theoretical sports knowledge and professional sports skills to students to cultivate the all-round sports talents needed by the country (Moy et al., 2016). College students majoring in sports have been engaged in sports for a long time; hence, they have unique cognitions and ways of thinking. College students majoring in sports are the vital support for a country to develop sports and high-end talents, leading the development of sports (Granero-Gallegos et al., 2019). In an effort to encourage talents for sports development, different countries have also issued many policies, and regulations that are conducive to the education of college students majoring in sports. With the ever-increasing trend of globalization, the overall national strength competition among countries has become particularly apparent. Both China and Malaysia attach great importance to the training and development of students majoring in sports, especially in competitive sports such as table tennis and badminton. However, while the cultivation of sports talents is valued, learning situation of students is also a big issue that cannot be ignored. The independent colleges and universities with sports majors have their unique professional advantages, providing much room for students majoring in sports (Erdvik et al., 2020). However, in this environment, students are more likely to have problems such as low enthusiasm and learning burnout, affecting the personal progress of students and the development of national sports (Potdevin et al., 2018; Trigueros et al., 2019).

Students majoring in sports are more likely to suffer from learning burnout than those in other majors. Hence, the impacts of learning motivation on learning burnout are analyzed to correct this lousy state, thereby stimulating the learning motivation of college students. With the development of Malaysia’s sports industry, a comparative analysis of the learning situation of Chinese and Malaysian students majoring in sports has practical significance in promoting the progress of China’s sports education. According to the theory of educational psychology, the *status quo* of learning motivation and learning burnout of college students majoring in sports is taken as the breakthrough point. Chinese and Malaysian college students majoring in sports are included as the research objects. The correlations between various dimensions of learning motivation and learning burnout are analyzed to determine the impact of the former on the latter. Colleges in Malaysia have autonomy and flexibility in the formulation of the syllabus of the physical education theory course. On the one hand, there is no national standard for physical education theory teaching in colleges in Malaysia, and there is no unified teaching goal and unified teaching task, which reflects full autonomy. On the other hand, there is no clear provision on the time arrangement of the syllabus of physical education theory course in colleges in Malaysia, and students can arrange the course time independently; teachers have no specified teaching materials and can choose their teaching contents, which reflects full flexibility. Malaysia’s college physical education curriculum attaches great importance to the personality development of students to enable them to obtain more strengthened social adaptability. The results obtained can deepen the understanding of the relationship between learning motivation and learning burnout and improve the theoretical system of learning burnout.

## Theoretical Basis and Research Methods

### Learning Motivation Analysis From an Educational Psychology Perspective

From the educational psychology perspective, motivation is the direct internal driving force to promote activities of an individual. Learning motivation means the direct internal driving force that promotes students to participate in learning activities. The prerequisite for the effective development of learning activities is learning motivation, which promotes students’ learning by enhancing their behaviors (Ulstad et al., 2018). If teachers and schools can help students form good learning motivation, they can fundamentally improve the learning outcomes of students.

Intrinsic motivation is the driving force for pursuing the pure pleasure of activities, while some activities in physical education may not be interesting (Xiang et al., 2017; Wang and Chen, 2019; Luo et al., 2020). Interest is generated and developed based on needs. When people are interested in some things or activities, they will present a positive state. They connect their positive emotions with these things and take the initiative to engage in these activities to continuously explore the nature of these things and activities and improve their knowledge literacy. Therefore, if students majoring in sports are forced to repetitively practice sports day after day rather than driven by their motivation, they will not integrate their goals or ideals during this process, and their internal motivation will eventually be worn away. Psychological research shows that learning motivation is not a single psychological activity. The generation of student learning activities is also driven by a dynamic system formed by various psychological factors (Huhtiniemi et al., 2019).

**FIGURE 1:**
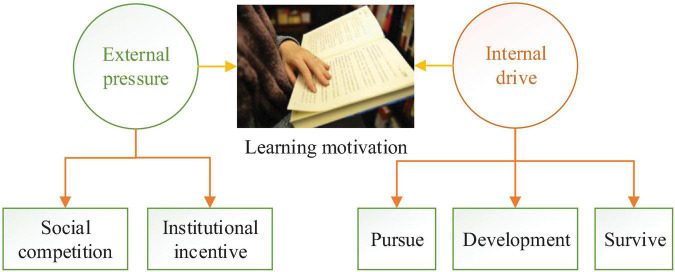
The generation mechanism of learning motivation of students.

Learning demand is an essential psychological factor produced by learning motivation, reflecting the demands of students for goals. According to its social meaning, learning motivation can be divided into noble and low-level motivations. The core of the noble learning motivation is altruism. Students associate their current learning with the interests of the country and society. In contrast, the core of low-level learning motivation is self-interested, self-centered, and derived from the interests of students. For the social purpose of social development and the personal purpose of self-development, once students recognize this learning demand, they will express their desire for learning (Amorose et al., 2016; Laxdal et al., 2020). When students combine the social value and personal value of learning organically, they can finally transform social requirements into their needs and then promote self-learning. The generation mechanism of learning motivation of students is shown in Figure 1.

Both educational practice and educational psychology experiments show that learning motivation can promote learning activities, stimulate the interest of students in learning, maintain a particular level of arousal, and lead to definite learning activities (Kalajas-Tilga et al., 2020). According to an American psychologist, Lazaros, students with a strong interest in learning show a greater advantage in academic performance compared with students with high intelligence. This finding also confirms that interest can promote students to learn more actively and consciously than intelligence. On the one hand, in the education process, teachers should focus on strengthening goals and clarifying learning goals and demands to students. On the other hand, teachers should also constantly try various novel training methods in the teaching process and plan interesting secondary classroom activities to stimulate the interest of students in learning, thereby enhancing their subjective initiative.

### Learning Burnout Causes of Students Majoring in Sports

**FIGURE 2:**
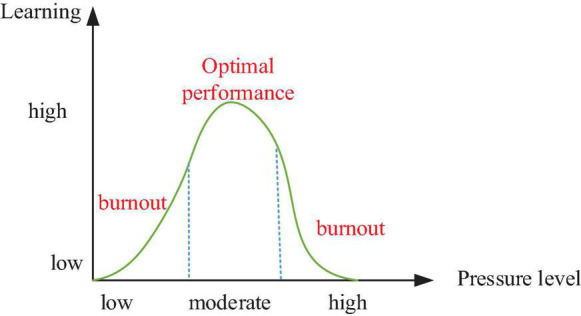
Correlation between learning burnout and pressure level.

The concept of “burnout” was proposed by Freudenberger, an American psychotherapist, in the 1970s. Burnout was defined as chronic fatigue, depression, and frustration. It was first investigated in the occupational field. With the gradual deepening of research on burnout, learning burnout is summarized as a kind of negative emotion caused by long-term learning pressure and workload load of students, leading to their loss of interest in learning activities (Ljubin-Golub et al., 2020). From a psychological perspective, when the pressure level exceeds the ability of an individual to withstand, and this individual has suffered from this problem for a long time, burnout emotions will appear eventually. Figure 2 shows the impact of pressure level changes on learning burnout. There is a significant positive correlation between academic pressure and learning burnout, that is, the greater the learning pressure is, the more students tend to adopt negative coping ways, which will lead to a higher level of learning burnout. Learning burnout has the following characteristics: (1) *Tired and sleepy*: The learning enthusiasm of students is reduced, and their emotional resources are exhausted. They easily feel depressed and cannot concentrate on learning. (2) *Lack of humanity*: Students are often rude to others due to emotional depression. They have a passive and negative attitude and do not recognize the evaluation and guidance of others. The aggressive behavior is an extreme manifestation of learning burnout. (3) *Low realizability*: Students will underestimate their learning achievements and even deny every effort they have made (Kroupis et al., 2019; Roohani and Dayeri, 2019; Lee et al., 2020).

Most college students majoring in sports have received formal physical training from an early age and can exert better professional skills in personally advantageous sports events. However, due to limited time and energy, their performance in cultural courses is generally lower compared to other college students. The public theoretical knowledge required in universities is the same for all majors; hence, the requirements for the learning ability and quality of college students are the same (Lee, 2019). In this case, students majoring in sports will inevitably fail to understand the knowledge in class. If they stay in this state for a long time, their theoretical scores and professional skills will appear differentiated. Consequently, it is hard for them to acquire a sense of achievement from classrooms, resulting in learning burnout.

According to relevant materials, the reasons for the learning burnout of students majoring in sports can be summarized into four aspects: society, school, family, and individual (Castillo et al., 2020). Social factors have the greatest impact on the negative emotions of students in the learning process, such as the bad social atmosphere, employment pressure, and the general public’s perception of sports majors. School factors include the sports curriculum setting, teaching management system, and the investment and innovation of sports resources. Family factors refer to the parenting ability and expectations of parents for their children. Individual factors of students include personal identification with the profession and self-control in the learning process (Ince, 2019; Kolomitro et al., 2020).

In general, learning burnout is the passive mental state of students toward learning. The learning burnout of college students majoring in sports is closely related to the lousy social atmosphere, employment pressure, education shortcomings of college students, and psychological factors of students. Among the above factors, employment pressure is an urgent reason for the learning burnout of students. Many students majoring in sports do not highly recognize their major and doubt whether their major can be recognized and needed by society. Therefore, society should encourage sports talents, affirm the value of sports talents, establish a fair and reasonable employment mechanism, and change the mentality of students majoring in sports that they are confused about the future. In the meantime, students should also correctly understand the current environment, maintain a sense of urgency when faced with employment pressure, and keep making breakthroughs in keeping with the trend of the times.

### College Students’ Self-Efficacy and Attribution of Success or Failure

**FIGURE 3:**
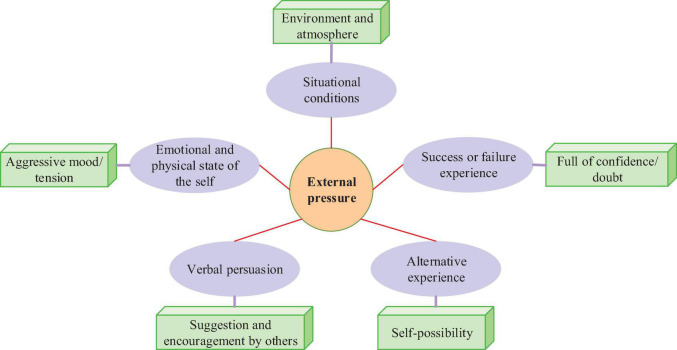
Five types of influencing factors of self-efficacy.

The self-efficacy theory was put forward by American psychologist Bandura from the perspective of social learning. Bandura adhered to some fundamental viewpoints of behaviorist psychology and emphasized the role of reinforcement in learning. In the meantime, Bandura also explored the psychological process and emphasized the mediating role of individual factors on behavior. Bandura believed that self-efficacy is affected by five types of factors: success or failure experience, vicarious experience, verbal persuasion, self-consciousness emotions and physical conditions, and situational constraints. The influencing factors of self-efficacy are shown in Figure 3. Successful experiences, such as achieving excellent grades and completing a project, will make college students full of confidence in the following learning and exploration tasks. In contrast, failure experiences, such as failing exams, will make students doubt themselves, personal abilities, and expected outcomes (Thompson et al., 2019). Vicarious experience refers to the ability of an individual to observe the behavior of others, thereby understanding the possibility of oneself. When students find that their classmates have a great chance of completing a learning task, they will think that the task is less difficult. Hence, they will increase their confidence in their ability to perform the learning task and are more willing to invest energy and effort (Jung et al., 2019; Bulfone et al., 2020). However, if most of the classmates of students classmates repeatedly fail when completing a learning task, students will invisibly increase their psychological pressure and subconsciously think that they are more likely to fail, resulting in learning burnout. The influence of verbal persuasion factors on self-efficacy refers to other people’s suggestions, persuasive suggestions, and self-exhortation. In particular, encouragement from classmates and teachers tends to make individuals more focused on learning goals and confident in completing tasks.

The correlation factors of learning burnout of college students are explored. The sense of self-efficacy in the learning activities of students can serve as an intermediary factor, which can affect the learning beliefs and behavioral activities of students. Students with a high sense of self-efficacy usually make greater efforts when they encounter obstacles in the learning process, while students with a low sense of self-efficacy are more likely to give up, and their attitude toward learning gradually becomes passive and slack (Alhadabi and Karpinski, 2020).

**FIGURE 4:**
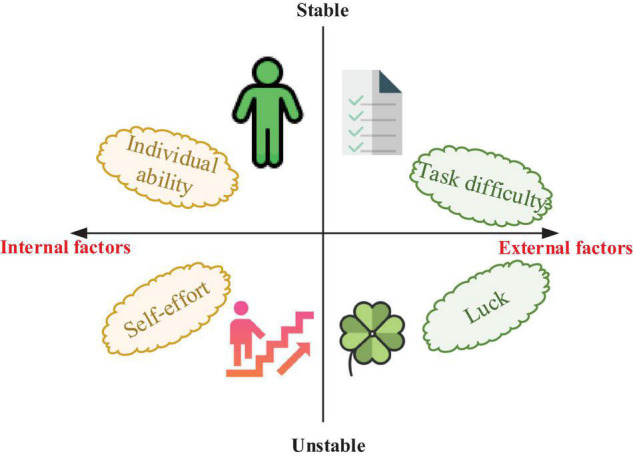
Factors of learning obstacles of students based on attribution theory.

The learning burnout problems of students are analyzed from the perspective of attribute theory. The correlation factors of learning obstacles of students are summarized in Figure 4. If students attribute the learning difficulties they encountered to the external environment, they will think that their burnout behaviors are irrelevant to individual factors, and they are not responsible for the consequences of their behaviors. On the contrary, if students attribute the difficulties encountered in learning to the individual factors, they will be more willing to actively solve the problem and take the initiative to take responsibility; as a result, learning burnout rarely occurs (Ardura and Galán, 2019; Ayllón et al., 2019). Attributing learning results to internal factors is conducive to future learning and positive learning motivation of students. If students attribute success to their abilities, they will think that success is due to their strong abilities and talents, which is not conducive to motivating their learning motivation. If students attribute failure to their abilities, they will think that failure occurs due to their lack of ability, and their self-confidence will be greatly frustrated, which will negatively affect students.

### Survey Objects and Research Methods

The research objects are Chinese and Malaysian college students majoring in sports. The Physical Education College of Zhengzhou University, China, and the University of Malaya, Malaysia, are selected. The learning motivation and learning burnout of students majoring in sports in the two countries are compared through questionnaire surveys. Besides, Chinese students majoring in sports are taken as examples to analyze the impact of learning motivation on learning burnout.

The learning motivation of college students is evaluated using the *Learning Motivation Scale* compiled by Tian Lan et al. This scale comprises 34 items, such as interest in knowledge (11 questions), ability pursuit (8 questions), reputation acquisition (7 questions), and altruistic orientation (8 questions) dimensions, scored using the 5-point Likert Scale. Specifically, “very consistent” is scored five points, “consistent” is scored four points, “neither consistent nor inconsistent” is scored three points, “inconsistent” is scored two points, and “very inconsistent” is scored one point. The interest in knowledge dimension exhibits the motivation of college students to develop their professional interests and obtain a pleasant experience. The ability pursuit dimension reveals the learning motivation of college students to improve their problem-solving abilities through learning, thereby increasing their employment competitiveness. The reputation acquisition dimension indicates the motivation of college students to expand their reputation through learning. The altruistic orientation dimension reflects the motivation of college students to be worthy of teacher training, help others, and contribute to society in the future. Earning burnout of college students is evaluated using the Learning Burnout Scale for College Students compiled by Lian Rong et al. This scale contains 20 items, such as depression (8 questions), misconduct (6 questions), and low sense of achievement (5 questions) dimensions. Similarly, its scoring method adopts the 5-point Likert Scale as well. The higher the score, the higher the burnout degree. Tests prove that the consistency reliability coefficients (Cronbach’s alpha) of the two scales are greater than 0.85, and the structural validity coefficients Kaiser-Meyer-Olkin (KMO) are greater than 0.70.

Four hundred students majoring in sports education and sports economic management in the second to the fourth year were randomly selected from the Sport School of Zhengzhou University and the University of Malaya Centre for Sport and Exercise Sciences, with 200 students from each university. The questionnaires were distributed and recovered online. This investigation was approved by the ethics committee. One hundred and ninety-five valid questionnaires were returned from the Sport School of Zhengzhou University, and 192 valid questionnaires were returned from the University of Malaya Centre for Sport and Exercise Sciences. The Excel and SPSS 21.0 statistical software are utilized to process the questionnaire data. Statistical methods, such as descriptive statistics and correlation analysis, are employed to analyze college students’ learning motivation, learning burnout, and the correlation between the two.

## Results

### Overall Description of Learning Motivation of Chinese and Malaysian College Students Majoring in Sports

**FIGURE 5:**
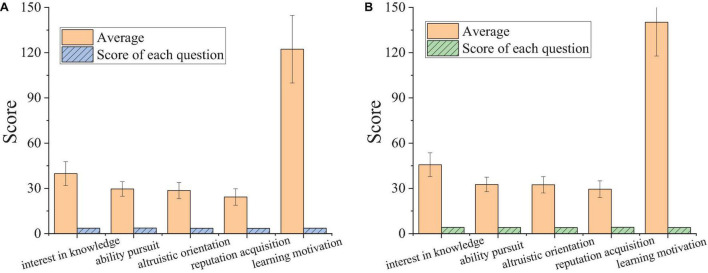
Descriptive statistics for the learning motivation of Chinese and Malaysian college students majoring in sports. **(A)** Sport School of Zhengzhou University. **(B)** Center for Sport and Exercise Science University of Malaya.

Descriptive statistics are adopted to analyze the learning motivation of students from the Sport School of Zhengzhou University and the University of Malaya Centre for Sport and Exercise Sciences, thereby better understanding the learning motivation of Chinese and Malaysian college students majoring in sports. The results are presented in Figure 5.

As shown in Figure 5, the total learning motivation scores of students from the Physical Education College of Zhengzhou University and the University of Malaya Centre for Sport & Exercise Sciences are 122.3 ± 22.4 and 140.2 ± 23.6, respectively, and their average scores of each question are 3.60 and 4.07. There is a significant difference between the two groups (*P* < 0.05). Overall, the learning motivation of Malaysian college students majoring in sports is slightly stronger than Chinese college students. The average scores of Chinese students majoring in sports in the four dimensions in descending order are: interest in knowledge, ability pursuit, altruistic orientation, and reputation acquisition; the average scores of Malaysian students majoring in sports in the four dimensions in descending order are: reputation acquisition, interest in knowledge, ability pursuit, and altruistic orientation. These data suggest that for Chinese college students majoring in sports, internal learning motivation has a stronger impact on learning motivation than external learning motivation. Besides, interest in knowledge has greater impacts than ability pursuit, and altruistic orientation has greater impacts than reputation acquisition. For Malaysian college students majoring in sports, reputation acquisition in external learning motivation has more prominent impacts on learning motivation, followed by the interest in knowledge.

Internal and external motivation can be combined to inspire the learning motivation of college students majoring in sports. First, the teaching methods should be enriched to stimulate the interest of students in learning and improve teaching quality so that students can acquire useful knowledge from the classrooms. Colleges and universities should also strengthen students’ ideological education and improve their enthusiasm and self-confidence in studying this major.

### Overall Description of Learning Burnout of Chinese and Malaysian College Students Majoring in Sports

**FIGURE 6:**
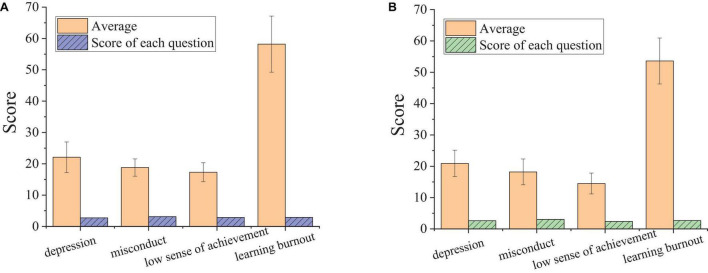
Descriptive statistics for learning burnout of Chinese and Malaysian college students majoring in sports. **(A)** Sport School of Zhengzhou University. **(B)** Center for Sport and Exercise Science University of Malaya.

Many factors can affect the learning burnout of students. The characteristics of different majors will also affect learning burnout. Therefore, the learning burnout of students majoring in sports is somewhat different from that of ordinary college students. Figure 6 displays the descriptive statistics for learning burnout of students from the Sport School of Zhengzhou University and the University of Malaya Centre for Sport and Exercise Sciences.

As shown in Figure 5, Chinese and Malaysian college students majoring in sports have learning burnout. The total learning burnout scores of students from the Sport School of Zhengzhou University and the University of Malaya Centre for Sport and Exercise Sciences are 58.2 ± 8.95 and 53.6 ± 7.34. There is a significant difference between the two groups (*P* < 0.05). Compared with Malaysian college students, the learning burnout of Chinese college students majoring in sports is more severe, mainly reflected in the two dimensions of depression and a low sense of achievement. In contrast, the difference between the two countries in terms of misconduct is not significant. The reason is that the employment pressure of college students majoring in sports in Malaysia is slightly lower than that in China, and Malaysian students have more freedom in employment choices. Besides, the society of Malaysia has a higher recognition of students majoring in sports, so that students of this major have a higher sense of achievement.

Regarding the learning burnout problems of students majoring in sports, colleges and universities should consider the mental health education of college students, correct the wrong learning cognition, improve the learning methods of students, and impart successful experiences. Colleges and universities can help students overcome learning burnout emotions through personal consultation and special mental health education. Teachers should adopt heuristic teaching models to continuously enrich the teaching content, enhance the charm of the teaching process, and enable students to participate in learning actively. In the meantime, teachers should comprehensively consider the connection between the theoretical knowledge and practice of the sports major so that knowledge can be valued in practice. Finally, students should emphasize the training of will and self-construction of personality. They should first improve their tolerance for learning burnout and improve their ability to overcome difficulties. In the meantime, they should actively correct unreasonable cognitions, control emotions through various means, and maintain learning enthusiasm.

### Correlation Analysis Between Learning Motivation and Learning Burnout of College Students Majoring in Sports

Motivation is always connected to activities. Motivations generated by individuals in different activities are also different. Learning motivation is the internal driving force for students to pursue their learning goals and the fundamental motivation for students to start and participate in learning activities. Learning burnout refers to a phenomenon of negative emotions about learning. Here, Chinese college students majoring in sports are taken as examples to explore the correlation between learning motivation and learning burnout. According to the survey results above, the total learning motivation score of Chinese college students majoring in sports is 122.3 ± 22.4, and their total learning burnout score is 58.2 ± 8.95. The correlation analysis results of the two are presented in Table 1. Furthermore, the correlations between the four dimensions of learning motivation and the three dimensions of learning burnout are analyzed, and the results are shown in Table 2.

**TABLE 1:** Correlation analysis between learning motivation and learning burnout.

Correlation		Learning motivation	Learning burnout
Learning motivation	Pearson correlation	1	−0.477**
	Significance	–	0.000
Learning burnout	Pearson correlation	−0.477**	1
	Significance	0.000	–

**There is a significant correlation at the0.01 level (bilateral).

**TABLE 2:** Correlation analysis between various dimensions of learning motivation and learning burnout.

Correlation		Learning burnout	Depression	Misconduct	Low sense of achievement
Learning motivation	Pearson correlation	−0.477**	−0.244**	−0.372**	−0.693**
	Significance	0.000	0.000	0.000	0.000
Interest in knowledge	Pearson correlation	−0.552**	−0.311**	−0.456**	−0.712**
	Significance	0.000	0.000	0.000	0.000
Ability pursuit	Pearson correlation	−0.422**	−0.215**	−0.305**	−0.605**
	Significance	0.000	0.000	0.000	0.000
Altruistic orientation	Pearson correlation	−0.435**	−0.243**	−0.331**	−0.592**
	Significance	0.000	0.000	0.000	0.000
Reputation acquisition	Pearson correlation	−0.261**	–0.052	–0.210	−0.388**
	Significance	0.000	0.398	0.022	0.000

**There is a significant correlation at the 0.01 level (bilateral).

Overall, learning motivation and learning burnout share a significantly negative correlation at the 0.01 level. In terms of each dimension, except for the dimensions of reputation acquisition, misconduct, and a low sense of accomplishment that share significantly negative correlations at the levels of 0.05 and 0.01, other dimensions of learning motivation (interest in knowledge, ability pursuit, and altruistic orientation) and learning burnout have a significantly negative correlation at the 0.01 level. In summary, the learning motivation and learning burnout of college students majoring in sports are negatively correlated. In other words, the stronger the learning motivation, the weaker the learning burnout; on the contrary, the weaker the learning motivation, and the stronger the learning burnout.

## Discussion

Sports is a unique education major for cultivating all kinds of sports talents. It imparts students of sports theoretical knowledge and technical skills to develop talents for national sports development. Sports is different from other majors. Students generally suffer from learning burnout. The main causes of learning burnout are analyzed from the perspective of educational psychology, which includes social factors, school factors, family factors, and personal factors. Besides, the impact of learning motivation on learning burnout is theoretically analyzed to solve the problem of learning burnout of college students. With Chinese and Malaysian students majoring in sports as the research object, the learning status of students majoring in sports in different countries is explored, and the impact of learning motivation on learning burnout is further analyzed.

Students majoring in sports education and sports economic management in the second to the fourth year were randomly selected from the Physical Education College of Zhengzhou University and the University of Malaya Centre for Sport & Exercise Sciences. The questionnaires were distributed and recovered online to investigate the learning motivation and learning burnout of college students. Besides, the learning burnout of Chinese college students majoring in sports is also more prominent, mainly reflected in the two dimensions of depression and a low sense of achievement. Correlation analysis reveals a negative correlation between learning motivation and learning burnout of college students majoring in sports. Therefore, teachers need to clarify the needs of students in the teaching process, and keenly perceive the changes of such needs, to promote their interest in the taught subjects, meet the basic knowledge-seeking needs of students, and stimulate the learning motivation of students. Students should establish a correct outlook on employment and face learning pressure and social pressure with a positive attitude. Colleges and teachers should scientifically adjust curriculum settings and guide students to learn independently. Society should also attach importance to sports talents, increase the recognition of sports majors, and enhance the learning enthusiasm and self-confidence of students. The learning motivation of students can be stimulated, and their learning burnout in the learning process can be alleviated through the joint efforts of all parties. Serious burnout affects the autonomous learning ability of college students. For the existing problems, corresponding effective measures are put forward to adjust the phenomenon of college students’ burnout and reduce the degree of burnout, which is conducive to the improvement of learning efficiency and academic achievement of college students.

## Conclusion

Overall, the learning motivation of Malaysian college students majoring in sports is slightly stronger than Chinese students. The lower the students’ self-efficacy is, the easier they feel learning burnout. There is a negative correlation between learning motivation and learning burnout of students majoring in sports. The stronger the learning motivation is, the weaker the students’ learning burnout is. This exploration provides a theoretical basis and practical reference value for dealing with the learning burnout of students and improving their learning enthusiasm to some extent. Some research achievements have been made, but there are also some deficiencies. Due to the limitation of capital and time, the selection range of sample size is limited. If the scope of sample size can be further expanded, the research results may be more typical, representative, and popularized. Only the questionnaire is adopted as the research method, and the results are easy to be affected by the samples. If the subjects do not fill in the questionnaire completely according to their true intention, it will greatly affect the research results. In the follow-up research, a combination of multiple research methods can be used to obtain the data.

## Data Availability Statement

The raw data supporting the conclusions of this article will be made available by the authors, without undue reservation.

## Ethics Statement

The studies involving human participants were reviewed and approved by University of Malaya Ethics Committee. The patients/participants provided their written informed consent to participate in this study. Written informed consent was obtained from the individual(s) for the publication of any potentially identifiable images or data included in this article.

## Author Contributions

All authors listed have made a substantial, direct, and intellectual contribution to the work, and approved it for publication.

## Conflict of Interest

The authors declare that the research was conducted in the absence of any commercial or financial relationships that could be construed as a potential conflict of interest.

## Publisher’s Note

All claims expressed in this article are solely those of the authors and do not necessarily represent those of their affiliated organizations, or those of the publisher, the editors and the reviewers. Any product that may be evaluated in this article, or claim that may be made by its manufacturer, is not guaranteed or endorsed by the publisher.
